# Hyperglycaemia-induced resistance to Docetaxel is negated by metformin: a role for IGFBP-2

**DOI:** 10.1530/ERC-16-0095

**Published:** 2016-11-21

**Authors:** K M Biernacka, R A Persad, A Bahl, D Gillatt, J M P Holly, C M Perks

**Affiliations:** 1 IGFs & Metabolic Endocrinology GroupSchool of Clinical Sciences, Learning & Research Building, Southmead Hospital, Bristol, UK; 2 Department of UrologySouthmead Hospital, Bristol, UK; 3 Department of Clinical OncologyBristol Haematology and Oncology Centre, University Hospitals Bristol, Bristol, UK

**Keywords:** hyperglycaemia, metformin, chemotherapy, prostate cancer, Docetaxel

## Abstract

The incidence of many common cancers varies between different populations and appears to be affected by a Western lifestyle. Highly proliferative malignant cells require sufficient levels of nutrients for their anabolic activity. Therefore, targeting genes and pathways involved in metabolic pathways could yield future therapeutics. A common pathway implicated in energetic and nutritional requirements of a cell is the LKB1/AMPK pathway. Metformin is a widely studied anti-diabetic drug, which improves glycaemia in patients with type 2 diabetes by targeting this pathway. We investigated the effect of metformin on prostate cancer cell lines and evaluated its mechanism of action using DU145, LNCaP, PC3 and VCaP prostate cancer cell lines. Trypan blue dye-exclusion assay was used to assess levels of cell death. Western immunoblotting was used to determine the abundance of proteins. Insulin-like growth factor-binding protein-2 (*IGFBP-2*) and *AMPK* genes were silenced using siRNA. Effects on cell morphology were visualised using microscopy. *IGFBP-2* gene expression was assessed using real-time RT-PCR. With DU145 and LNCaP cells metformin alone induced cell death, but this was reduced in hyperglycaemic conditions. Hyperglycaemia also reduced the sensitivity to Docetaxel, but this was countered by co-treatment with metformin. LKB1 was required for the activation of AMPK but was not essential to mediate the induction of cell death. An alternative pathway by which metformin exerted its action was through downregulation of IGFBP-2 in DU145 and LNCaP cells, independently of AMPK. This finding could have important implications in relation to therapeutic strategies in prostate cancer patients presenting with diabetes.

## Introduction

Prostate cancer (PCa) is the most common non-cutaneous cancer in men, and it accounts for the second highest cause of mortality among men in the US after lung cancer (Siegel *et al*. 2012).

The global obesity and associated diabetes epidemics have been linked with an increase in the development and/or progression of a number of cancers. In general, epidemiology has revealed an inverse correlation between diabetes and risk of developing PCa considered to potentially be due to the insulin resistance occurring in diabetes being accompanied by reduced levels of testosterone that is one of the main drivers for PCa progression. Men with diabetes are however at a greater risk of mortality from prostate cancer (Bonovas *et al*. 2004, Kasper & Giovannucci 2006). Therefore, new ways are needed to improve response to current treatment and to decrease mortality associated with diabetes and PCa. There is currently a lot of interest in metformin, a drug commonly used to treat diabetes mellitus, that became a standard treatment after other members of the biguanides family, phenformin and buformin, which were discontinued due to increased risk of lactic acidosis (Kolata 1979, Bailey & Turner 1996, Witters 2001).

Beneficial effects of metformin have been described with an apparent decrease in the incidence of several cancers. Patients with diabetes had a significantly lower risk of developing colon cancer with metformin use (Tseng 2012). Metformin use has also been associated with reduced prevalence of breast (Libby *et al*. 2009) and pancreatic cancers (Currie *et al*. 2009). Studies of men with localised prostate cancer showed that the use of metformin was associated with an improvement in cancer-specific survival and a decrease in the transition from androgen-sensitive to castration-resistant PCa (CRPC) (Spratt *et al*. 2013). Other studies have also suggested a beneficial effect of metformin use in patients with diabetes in relation to cancer incidence (Murtola *et al*. 2008, Wright & Stanford 2009) and improved survival in prostate cancer patients (He *et al*. 2011).

The anticancer effects of metformin could be mediated by a canonical pathway involving the activation of AMP-activated protein kinase (AMPK). In response to low energy levels or cellular stress, a conformational change is induced in the AMPKγ regulatory subunit that results in the exposure of the AMPKα catalytic subunit to phosphorylation of threonine residue (Thr-172) by liver kinase B1 (LKB1/STK11) (Shackelford & Shaw 2009). Activated AMPK can then control cell growth and proliferation by regulating the activity of the mammalian target of rapamycin (mTOR) (Wullschleger *et al*. 2006). This results in an inhibition of protein synthesis and proliferation. Whether the presence of LKB1 is required for the actions of metformin are however not clear. In some studies, metformin was unable to inhibit cell growth in LKB1-negative HeLa cells but did reduce growth in LKB1-positive breast cancer (MCF7) and normal breast epithelial cells (MCF10a) or in prostate cancer cells (PC3) (Zakikhani *et al*. 2006). In contrast to this study, Algire and coworkers showed that metformin was able to reduce tumour growth induced by a high-fat diet and high glucose regardless of *LKB1* expression *in vivo* (Algire *et al*. 2011).

In our study, we present data showing that metformin can act in an LKB1-AMPK-independent way to induce prostate cancer cell death and counter hyperglycaemia-induced chemoresistance and that these effects involve the regulation of IGFBP-2.

## Materials and methods

### Reagents

All chemicals, unless otherwise stated, were purchased from Sigma. Compound C was purchased from Millipore. All siRNAs were purchased from Qiagen and the transfection reagent (SR-1003-04) from Synvolux Therapeutics (Groningen, The Netherlands). Foetal bovine serum (FBS) was purchased from Invitrogen, DMEM-25 mM glucose (BF-709) and DMEM-5 mM glucose (BF-708), RPMI-1640, penicillin–streptomycin solution and l-glutamine were bought from Lonza (Basel, Switzerland). Human, recombinant non-glycosylated IGFBP-2 was obtained from GroPep (Thebarton, Australia).

### Cell culture and imaging

Prostate cancer cell lines: androgen-independent DU145, PC3 and androgen-dependent LNCaP and VCaP were purchased from ATCC. DU145, PC3 and VCaP cells were grown in DMEM growth media supplemented with 10% v/v foetal bovine serum (FBS), penicillin–streptomycin (50 IU/mL) and 1% v/v l-glutamine solution (2 mM), and LNCaP cells were cultured in RPMI-1640 growth media supplemented with 10% v/v FBS and antibiotics. Cells were maintained in a humidified 5% carbon dioxide atmosphere at 37°C. For all experiments, cell lines were seeded in 5 mM glucose growth media (GM) for 24 h and next transferred to serum-free media (SFM) containing either 5 mM or 25 mM glucose, supplemented with sodium bicarbonate (1 mg/mL), bovine serum albumin (0.2 mg/mL) and transferrin (0.01 mg/mL). After a further 24 h, the cells were dosed with Docetaxel (0–60 nM), Metformin (0–10 mM) and Compound C (2 µM) according to the respective figure legends. Dead cells were assessed using trypan blue cell counting as described previously (Thomas *et al*. 2010). Images were taken with a 10× objective on Zeiss Axiovert microscope using Moticam 5.0 MP camera (Hong Kong, China).

### Silencing AMPKα and IGFBP-2 using siRNA technology

Cells were seeded in 24-well or 6-well plates with cell a density of 0.04 × 10^6^ cells/well and 0.3 × 10^6^ cells/well respectively, in 5 mM glucose GM in the presence or absence of *AMPKα1* siRNA (target sequence 5′-CCGAAGTCAGAGCAAACCGTA-3′), *AMPKα2* siRNA (target sequence 5′-CCCACGATATTCTGTACACAA-3′) and IGFBP-2 siRNA (target sequence CCCGGAGCAGGTTGCAGACAA) at a concentration of 75 nM for *AMPK* and 25 nM for IGFBP-2 or with a random sequence negative control siRNA (NSsiRNA). The next day GM was switched to SFM for a further 24 h before dosing with drugs of interest for another 24 h. Cell death was assessed as described in Thomas *et al*. (2010), and IGFBP-2 and AMPK abundance were monitored using Western immunoblotting.

### Western immunoblotting

Equal amount of protein lysates estimated by BCA protein assay (Thermo Fisher Scientific, 23225) or equal volume of concentrated cell conditioned media were separated on a 8–12% SDS-PAGE gels and transferred to Hybond N+ nitrocellulose membranes (Amersham, RPN119B). Non-specific binding sites on the membranes were blocked for a minimum of 2 h with 5% w/v milk or 3% w/v bovine serum albumin (BSA) for non-phosphorylated and phosphorylated proteins respectively in Tris-buffered saline (TBS)/2% v/v Tween (TBS/T) before overnight (4°C) probing with antiserum against phospho-AMPKα (Thr172) (1:1000: Cell Signalling 2535), LKB1 (1:1000 Santa Cruz sc-32245), IGFBP-2 (1:1000: Santa Cruz sc-6001), GAPDH (1:5000 Millipore MAB 374), tubulin (1:5000: Thermo Fisher Scientific, MZ05829) or β-actin (1:10,000 Sigma-Aldrich A5441). Secondary anti-goat antibody was used at 1:2000 dilutions for IGFBP-2, anti-rabbit for Phospho-AMPKα at 1:2000 dilution or anti-mouse antibody (1:5000) for LKB1, tubulin, β-actin or GAPDH. Peroxidase binding was visualised by enhanced chemiluminescence and detected using ChemiDoc XRS+ System and analysed using Image Lab Software (BioRad, 170-8265). Western immunoblots were quantified using BioRad Quantity One 4.6.5 1-D Analysis Software.

### Quantitative RT PCR

Total RNA from cells seeded at 1 × 10^6^ cells and cultured in T25 flasks were extracted using TRIzol reagent (Invitrogen) according to the manufacturer’s instructions. 2 μg of total RNA was used for cDNA synthesis with random hexamers. Real-time PCR was carried out using Green JumpStart SYBR (Sigma, H5041) and an ABI StepOne Plus Realtime PCR System (Applied Biosystems, 4376600). Reactions were run in duplicate in three independent experiments. Expression data were normalised to the geometric mean of a housekeeping gene (*18S*) to control the variability in expression levels and were analysed using the 2^−∆∆CT^ method. PCR primers were designed using OligoPerfect online software from Qiagen under consideration of the special design criteria for real-time RT-PCR primers, spanning the junction between exons. *IGFBP-2* primers for PCR were used with the following sequences: forward 5′-CCTCAAGTCGGGTATGAAGG-3′ and reverse 5′-ACCTGGTCCAGTTCCTGTTG-3′ (primer size 162 bp). *18S* primers with the following sequences were used for normalization: forward 5′-GATGTAGTTGCTTGGGACCCA-3′ and reverse 5′-TGGAGATAACACTCTAAGCATAACTAAAGGT-3′ (primer size 140 bp) (both purchased from Thermo Scientific). Melt curves were performed for each RT-PCR analysis to ensure that no non-specific amplification was occurring (data not shown).

### Statistical analysis

Data were analysed with SPSS 13.0 for Windows using one-way ANOVA followed by least significant difference (LSD) *post hoc* test. A statistically significant difference was considered to be present at *P* < 0.05.

## Results

### Effects of metformin on cell death of PCa cells

**Figure 1:**
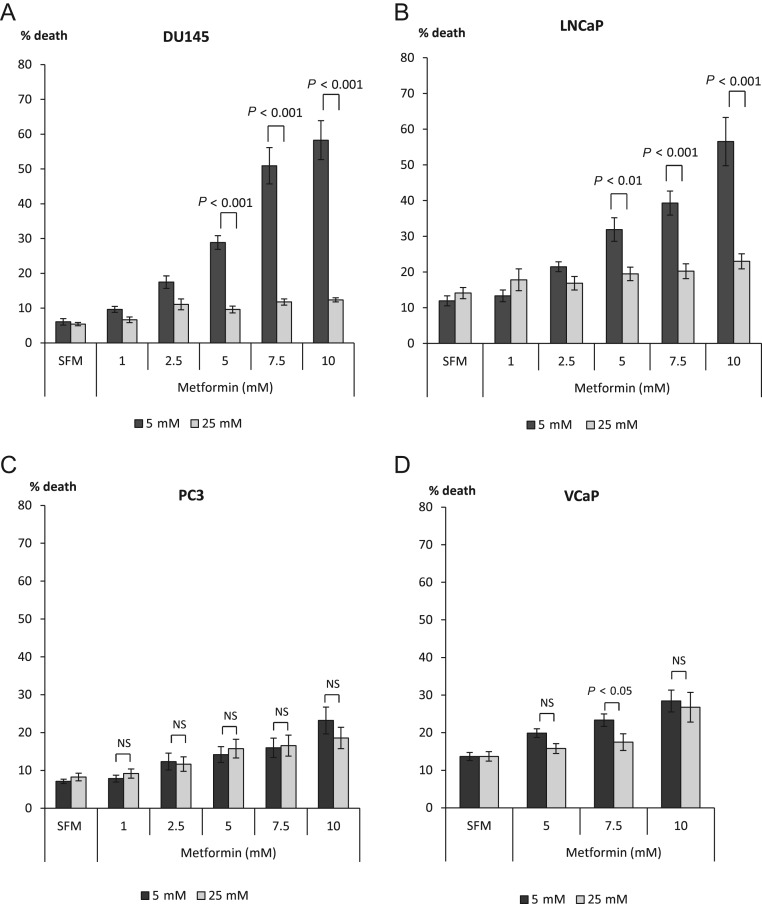
Dose response to metformin (0–10 mM) in PCa cell lines. Graphs show changes in % cell death in response to metformin. (A) DU145 cells were exposed to metformin in different glucose concentrations (5–25 mM). Cells were plated in six-well dishes (0.2 × 10^6^ cells/well or 0.3 × 10^6^ for LNCaP cells) in 5 mM glucose GM. The next day, media was switched to SFM containing different glucose concentrations (5 mM or 25 mM) for another 24 h. Cells were then dosed with metformin for a further 24 h. Dead cells were counted using trypan blue cell staining. (B) LNCaP, (C) PC3 and (D) VCaP cells were set up as described in Fig. 1A and levels of cell death were assessed using trypan blue staining. Graphs show mean of three experiments each repeated in triplicate.

The effect of metformin (1–10 mM) was examined in DU145, LNCaP, PC3 and VCaP PCa cells cultured in either euglycaemic (5 mM) or hyperglycaemic (25 mM) conditions (Fig. 1). With DU145, we found that metformin was able to induce a dose-dependent increase in cell death under euglycaemic conditions compared with hyperglycaemic conditions where cell death was not significantly affected by any dose of metformin. A statistically significant difference between glucose conditions was observed at 5 mM metformin: cell death was only increased by 4.2% (1.8-fold) in high glucose, compared with a 22.8% (4.8-fold) increase under euglycaemic conditions (Fig. 1A). A similar effect of metformin was seen in LNCaP cells, where we also observed an inability of metformin to induce cell death under hyperglycaemic conditions, whereas a dose-dependent increase in cell death could be seen under euglycaemic conditions. A 5 mM dose of metformin was able to induce a 20% (2.6 fold) increase of cell death in 5 mM glucose, compared with a 5.5% (1.4-fold) increase in hyperglycaemic conditions (Fig. 1B).

In contrast to the increased cell death observed in response to metformin treatment under euglycaemic conditions with DU145 and LNCaP cells, we found a moderate effect on cell death in PC3 and VCaP cells in response to metformin under either normal or high levels of glucose (Fig. 1C, D). Differences in the response to metformin in normal or high glucose conditions were observed at a dose of 5 mM with DU145 and LNCaP cells, but no significant differences in response to metformin were observed in either PC3 or VCaP cells.

**Figure 2:**
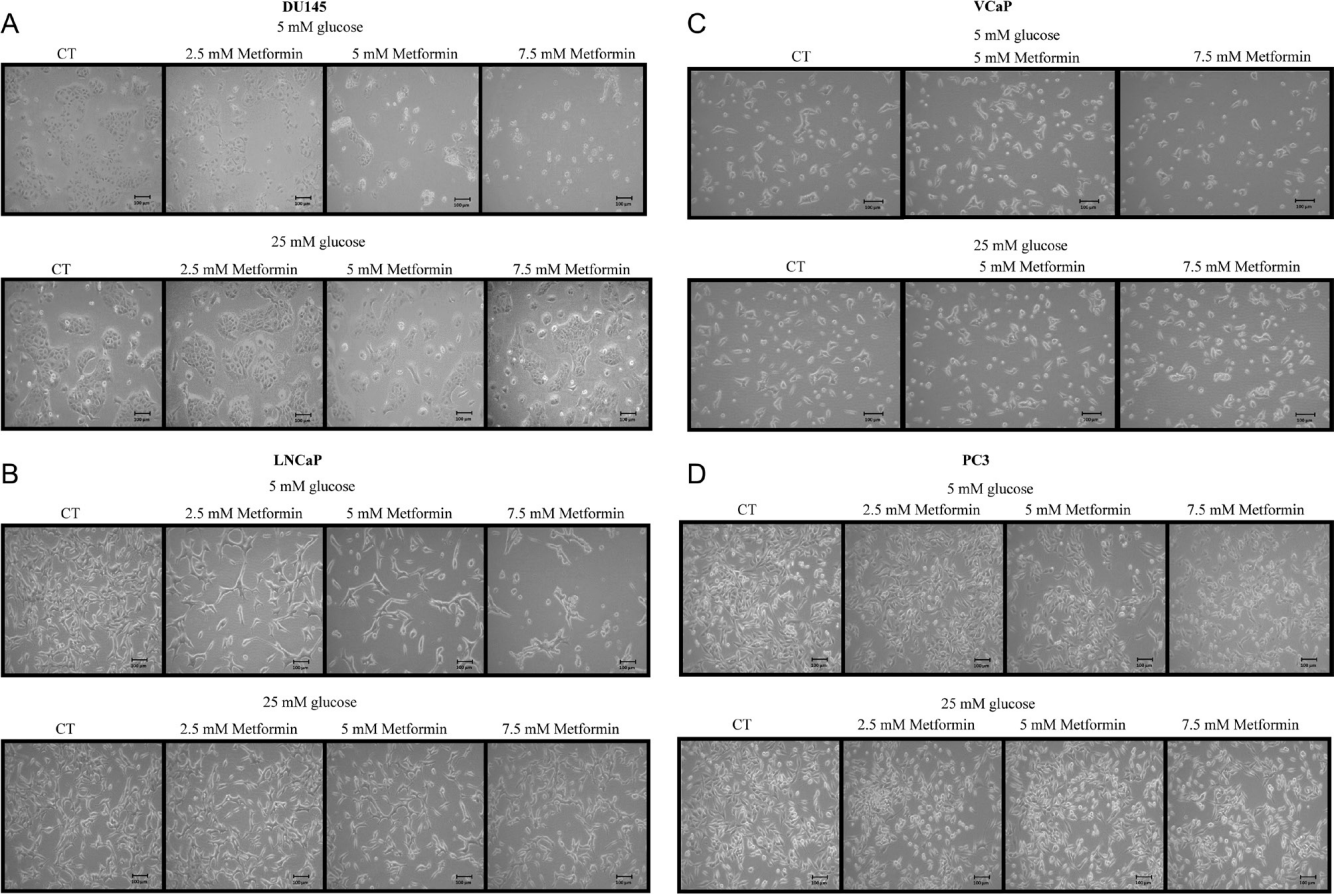
Changes in prostate cancer cell morphology treated with metformin (2.5–7.5 mM). (A) DU145 cells were plated in T-25 flasks (0.3 × 10^6^ cells/flask) in 5 mM glucose GM. The next day, media was switched to SFM containing different glucose concentrations (5 mM or 25 mM) for additional 24 h. Cells were then dosed with metformin for a further 24 h. Metformin induced a dose-dependent reduction in cell number and increased cell detachment in 5 mM but not in 25 mM glucose conditions. (B) LNCaP cells were set up as described in Fig. 2A and metformin induced a dose-dependent reduction in cell number in 5 mM but not in 25 mM glucose conditions. (C) VCaP cells were set up as described in Fig. 2A and metformin treatment induced some reduction in cell number in 5 mM but not in 25 mM glucose conditions. (D) PC3 cells were set up as described in Fig. 2A. Metformin did not alter cell number in either 5 mM or in 25 mM glucose conditions. All images were acquired under 10× magnification.

Figure 2A shows that treatment with metformin (2.5–7.5 mM) induced a significant reduction in DU145 cell number with cells becoming rounded and detached from the surface of the culture dish when cultured in 5 mM glucose conditions, but no such effect was observed in 25 mM glucose media. LNCaP cells responded similarly to increased concentrations of metformin, and a dose-dependent reduction in cell growth in normal glucose conditions was observed but not in high glucose-containing media (Fig. 2B). With VCaP cells, this pattern was observed only in the presence of 7.5 mM metformin in 5 mM glucose but not in 25 mM glucose (Fig. 2C). With PC3 cells, no differences in morphology were observed consistent with no effect of metformin on the induction of cell death (Fig. 2D).

**Figure 3:**
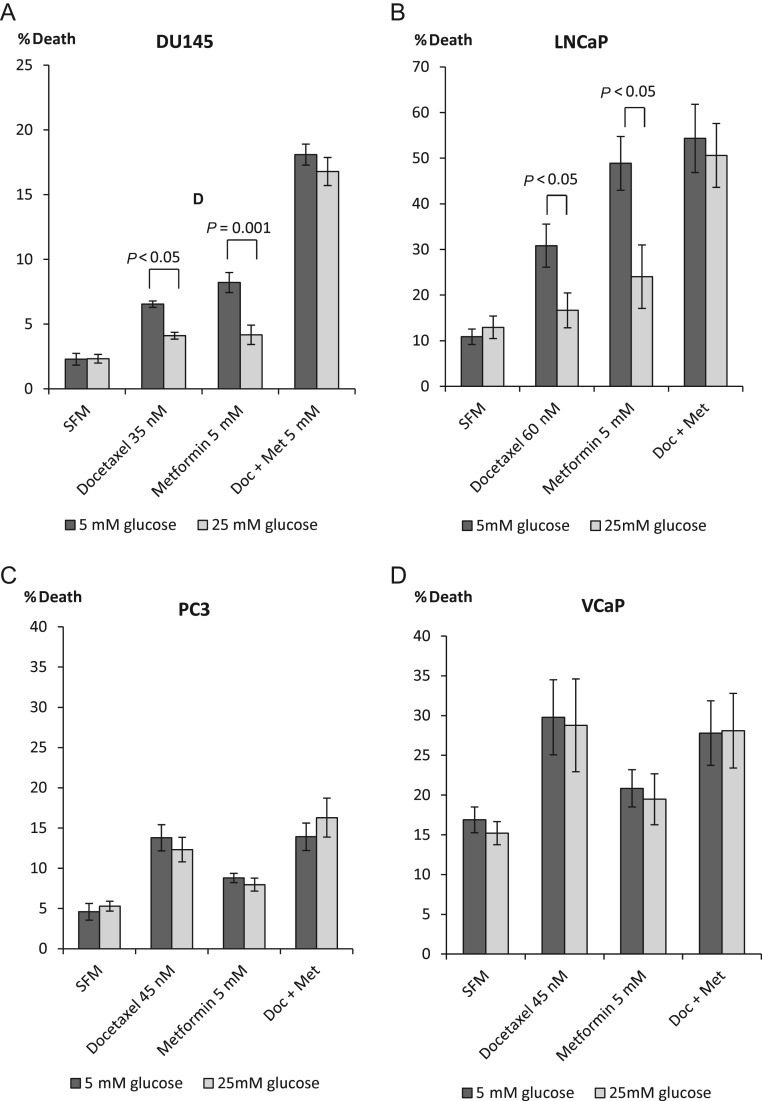
Changes in % cell death in response to single or combination treatment with Docetaxel (35–60 nM) and metformin (5 mM) in prostate cancer cell lines. (A) DU145 cells were set up as described in Fig. 1A and dosed with 35 nM Docetaxel, 5 mM metformin or with both for a further 24 h. (B) LNCaP cells were set up as described in Fig. 1A and dosed with 60 nM Docetaxel, 5 mM metformin or combined treatment for a further 24 h. (C) PC3 cells were set up as described in Fig. 1A and dosed with 45 nM Docetaxel, 5 mM metformin or both for a further 24 h. (D) VCaP cells were set up as described in Fig. 1A and treated with 45 nM Docetaxel, 5 mM metformin or both for another 24 h. For all figures, levels of % cell death were assessed using trypan blue cell counting. All graphs show mean of three experiments each repeated in triplicate.

We have published previously that hyperglycaemia confers survival against Docetaxel-induced cell death in DU145 and LNCaP but not PC3 PCa cell lines (Biernacka *et al*. 2013). Epidemiology studies have reported that patients taking metformin have decreased cancer incidence and/or improved cancer prognosis (Murtola *et al*. 2008, Wright & Stanford 2009, He *et al*. 2011). We then assessed the effect of metformin treatment in addition to Docetaxel. With DU145, we confirmed that hyperglycaemia conferred survival in response to Docetaxel: 4.2% (2.8-fold) increase in cell death in 5 mM vs 1.8% (1.8-fold) increase in 25 mM glucose conditions and to metformin alone: 5.9% (3.6-fold) in normal glucose in comparison to 1.9% (1.8-fold) increase in high glucose, but in combination, high glucose could no longer confer a protective effect against Docetaxel, and there was a synergistic increase in cell death in both levels of glucose. Docetaxel plus metformin treatment was effective in both glucose conditions and induced a 15.7% (7.6-fold) and 14.4% (7.3-fold) increase in cell death in 5 and 25 mM glucose respectively (Fig. 3A). A similar pattern was observed with LNCaP cells (Fig. 3B) where Docetaxel elevated the levels of dead cells by 20% (2.8-fold) in 5 mM glucose and only by 3.7% (1.3-fold) in 25 mM glucose and metformin alone induced a 38% (4.5-fold) increase in cell death in normal glucose compared with 11% (1.3-fold) in high glucose, whereas both drugs resulted in an additive effect of 44% (5-fold) and 37% (3.9-fold) elevated level of dead cells in 5 mM and 25 mM glucose conditions respectively. As described previously, there was no difference in response of PC3 and VCaP cells to a single treatment with Docetaxel or metformin with either normal or high levels of glucose. The combination treatment as anticipated did not induce any additional effect in relation to cell death (Fig. 3C and D).

**Figure 4:**
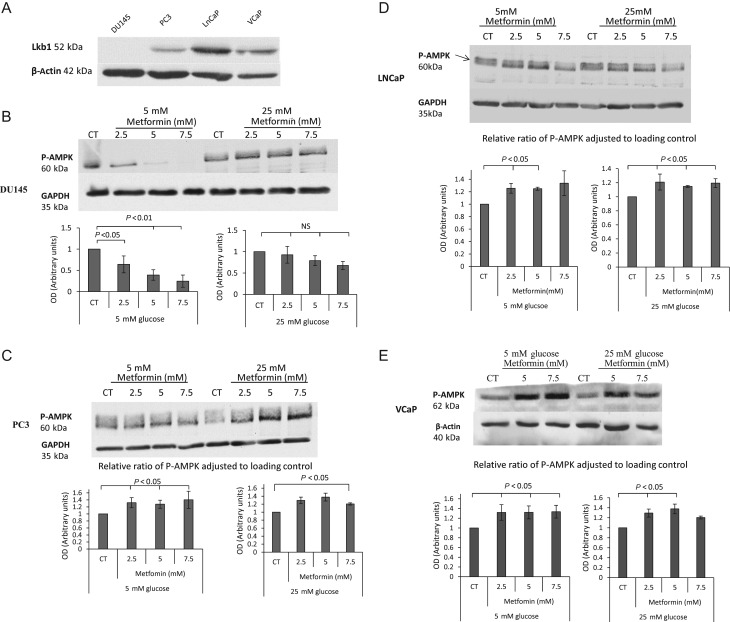
(A) DU145, PC3, LNCaP and VCaP PCa cell lines were screened for abundance of LKB1 protein. Cells were grown in growth media and whole-cell lysates were prepared and subjected to Western blotting. (B) Changes in p-AMPK levels after metformin (2.5–7.5 mM) treatment. DU145 cells were seeded in 5 mM glucose for 24 h, which was replaced with either 5 mM or 25 mM SFM for a further 24 h. Cells were then treated with metformin (2.5–7.5 mM) for 24 h and whole-cell lysates were prepared and subjected to Western blotting. 50 µg of protein were loaded onto a 10% gel. (C) Shows levels of p-AMPK in PC3 cells exposed to metformin (2.5–7.5 mM) treatment. Cells were set up as described in Fig. 4B. (D) Shows levels of P-AMPK in LNCaP cells exposed to metformin (2.5 and 5 mM) treatment. Cells were set up as described in Fig. 4B. (E) Shows levels of p-AMPK in VCaP cells exposed to metformin (5 and 7.5 mM) treatment. Each blot is representative of experiments repeated three times, and the densitometry shows the changes of p-AMPK adjusted to loading control for all experiments (*n* = 3).

Having observed the beneficial effect of metformin on chemotherapy-induced cell death negating the effect of high glucose, we investigated the mechanism underlying these responses. It has previously been reported that metformin downregulates the levels of IGF-IR in prostate cancer cells (Malaguarnera *et al*. 2014, Kato *et al*. 2015), but we assessed the abundance of IGF-IR by Western blotting and measured the levels of IGF-I and IGF-II by radioimmunoassay, and none of these variables were altered in the DU145 or LNCaP cells in these experiments (data not shown). Metformin activates AMPK, a central energy sensor of the cell, and this can be mediated by a separate kinase LKB1 (Zakikhani *et al*. 2006). We screened DU145, LNCaP, PC3 and VCaP PCa cell lines for LKB1 abundance in cell lysates. We could detect LKB1 protein in all cell lines except DU145 cells (Fig. 4A). Having identified the presence/absence of LKB1, we investigated the impact of metformin on the phosphorylation of its direct target – AMPK (Fig. 4B). As anticipated, with DU145, we did not observe the activation of AMPK in the presence of metformin due to the lack of LKB1. Interestingly, there was even a decrease in P-AMPK (Fig. 4B) in 5 mM glucose compared with no effect in 25 mM glucose conditions. We then examined PC3, LNCaP and VCaP cells, which are LKB1 positive and found that metformin could dose dependently activate AMPK in each of these cell lines in both glucose conditions (Fig. 4C, D and E respectively).

**Figure 5:**
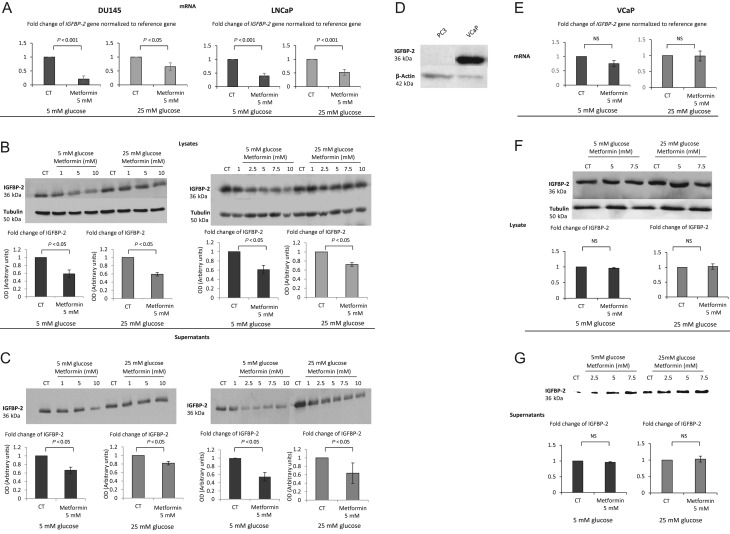
(A) Changes in mRNA levels of *IGFB-2* in response to 5 mM metformin treatment in either 5 mM or 25 mM glucose conditions. DU145 and LNCaP cells were seeded at 0.8 × 10^6^ cells/T-25 flasks and cultured as described in Fig. 1A, mRNA was extracted 24 h after dosing with metformin and Q-PCR was performed. Values of *IGFBP-2* gene expression were normalised to the housekeeping gene (*18S*) (*n* = 3). (B) Western immunoblots show changes in the abundance of IGFBP-2 from DU145 or LNCaP cell lysates respectively, exposed for 24 h to (1–10 mM) metformin in either 5 mM or 25 mM glucose. Cells were seeded at 0.5 × 10^6^ cells/T-25 flask and cultured as described in Fig. 1A and whole-cell lysates were collected and subjected to Western blot technique. Each blot is representative of experiments repeated three times, and the densitometry shows the mean changes (*n* = 3). (C) Western immunoblot shows changes in the abundance of IGFBP-2 from DU145 cell supernatants exposed for 24 h to (1–10 mM) metformin in either 5 mM or 25 mM glucose. Cells were set up as described in Fig. 1A, and conditioned media was collected and subjected to Western blot technique. Each blot is representative of experiments repeated three times and densitometry shows the mean changes at a representative dose of metformin (*n* = 3). (D) Western immunoblot shows changes in the abundance of IGFBP-2 in PC3 and VCaP cells. (E) Changes in mRNA levels of *IGFBP-2* in response to 5 mM metformin treatment in either 5 mM or 25 mM glucose conditions. VCaP cells were seeded at 0.8 × 10^6^ cells/T-25 flasks and cultured as described in Fig. 1A, mRNA was extracted 24 h after dosing with metformin and Q-PCR was performed. Values of *IGFBP-2* gene expression were normalised to the housekeeping gene (*18S*) (*n* = 3). (F) Western immunoblots show changes in the abundance of IGFBP-2 from VCaP cell lysates respectively, exposed for 24 h to (5–7.5 mM) metformin in either 5 mM or 25 mM glucose. (G) Western immunoblot shows changes in the abundance of IGFBP-2 from VCAP cell supernatants exposed for 24 h to (2.5–7.5 mM) metformin in either 5 mM or 25 mM glucose.

**Figure 6:**
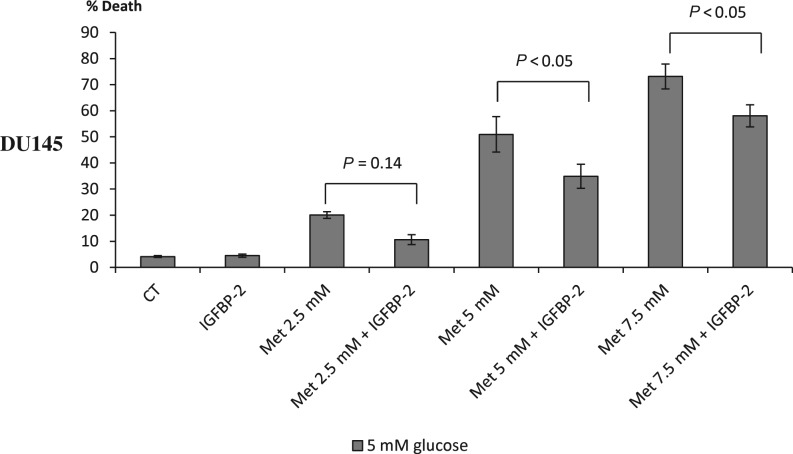
Changes in % cell death in response to treatment of metformin and/or IGFBP-2. DU145 cells seeded in six-well plates at 0.2 × 10^6^ cell/well with 5 mM glucose for 24 h. Cells were then transferred to 5 mM SFM and were treated for 24 h with 250 mM IGFBP-2 and/or 2.5–7.5 mM metformin. Cell death was assessed using trypan blue cell counting. Graph shows the mean of four experiments each repeated in triplicate.

We previously published that the protective effect of hyperglycaemia in response to Docetaxel was mediated by IGFBP-2 (Biernacka *et al*. 2013). As we had observed a similar protective effect of high glucose against metformin-induced cell death in DU145 and LNCaP cells, we then examined the influence of metformin on IGFBP-2. With both DU145 and LNCaP PCa cell lines, metformin was able to decrease mRNA levels of the *IGFBP-2* gene (Fig. 5A respectively). This decrease in gene expression resulted in a dose-dependent decrease in IGFBP-2 protein from whole cell lysates (Fig. 5B) as well as secreted into the conditioned media (Fig. 5C). The densitometry graphs show a statistically significant reduction in IGFBP-2 adjusted to loading control with a set dose of metformin (5 mM) compared with control in either euglycaemic or hyperglycaemic conditions (Fig. 5B and C inserts). As previously stated, in PC3 and VCaP cells, we did not observe any additional benefit of metformin in combination with chemotherapy. Levels of IGFBP-2 in PC3 cells are almost undetectable (Fig. 5D) and therefore we used VCaP cells as a negative control in investigating the associations between IGFBP-2 and metformin. We confirmed no change in mRNA level of the *IGFBP-2* gene in either 5 or 25 mM glucose conditions (Fig. 5E), no change in IGFBP-2 protein from whole cell lysates (Fig. 5F) or conditioned media (Fig. 5G). Having observed the beneficial effect of metformin in relation to elevated levels of cell death and its impact on IGFBP-2 protein and gene expression, we then assessed the effect of adding exogenous IGFBP-2, to counter the metformin-induced decrease, on metformin-induced cell death. Addition of exogenous IGFBP-2 significantly inhibited metformin (5 mM)-induced cell death of DU145 cells by 31.5% and 20.7% with 5 and 7.5 mM metformin respectively (Fig. 6).

**Figure 7:**
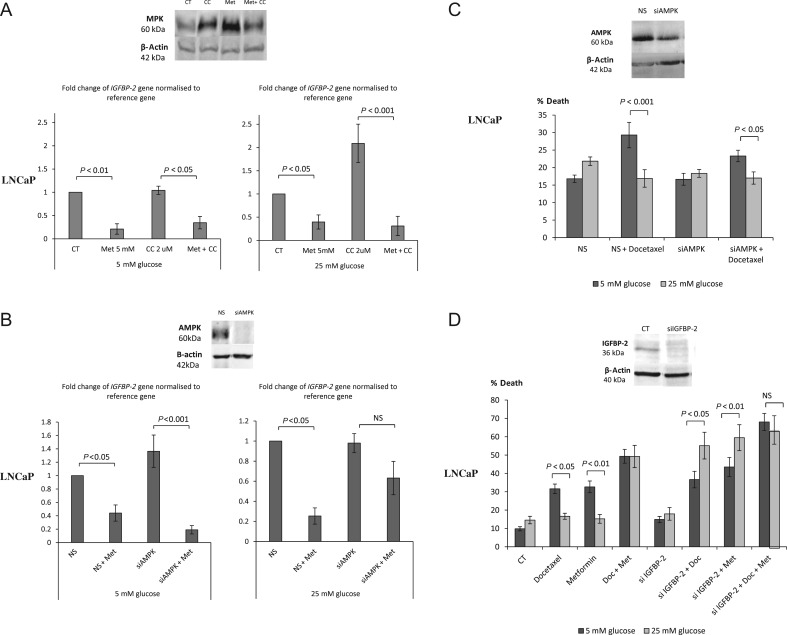
Changes in *IGFBP-2* gene expression in response to different treatments in either 5 mM or 25 mM glucose conditions. (A) LNCaP cells were set up as described in Fig. 5A and pre-treated with an AMPK inhibitor – Compound C (2 µM) for 1 h before dosing with 5 mM metformin for another 24 h. The mRNA was extracted and Q-PCR was performed. Values of *IGFBP-2* gene expression were normalised to the housekeeping gene (*18S*) (*n* = 3). Western blot insert shows successful inhibition of metformin-induced AMPK phosphorylation by Compound C. (B) LNCaP cells were transfected with 75 nM siRNA for α1 and α2 subunits of AMPK or 25 mM non-silencing control, whereas seeding in six-well plates (0.3 × 10^6^ cells/well) with 5 mM glucose and cultured as described in Fig. 1A. After 24 h treatment with 5 mM metformin, mRNA was extracted and Q-PCR was performed. Values of *IGFBP-2* gene expression were normalised to the house-keeping gene (*18S*) (*n* = 3). (C) Changes in % cell death of LNCaP cells silenced with 75 nM siRNA for *AMPK* or 25 nM non-silencing control, whereas seeding in six-well plates (0.3 × 10^6^ cells/well) with 5 mM glucose and cultured as described in Fig. 1A. After 24 h treatment with 60 nM Docetaxel for further 24 h, levels of cell death were assessed using trypan blue cell counting (*n* = 3). Western blot insert shows effective silencing of AMPK. (D) Changes in % cell death of LNCaP cells set up as described in Fig. 6C but silenced with 30 nM *IGFBP-2* siRNA or 25 nM non-silencing control and treated with 5 mM metformin or/and with 60 nM Docetaxel for 24 h. Cell death was assessed by counting using trypan blue (*n* = 3). Western blot insert shows effective silencing of IGFBP-2.

Having implicated IGFBP-2 in the effects of metformin on DU145 cells that lack LKB1, we then investigated the effect of metformin on LNCaP cells that unlike DU145 cells express LKB1. With LNCaP cells, metformin had the ability to phosphorylate AMPK (Fig. 4D) and was able to decrease *IGFBP-2* gene and protein expression (Fig. 5). We then inhibited AMPK using Compound C (CC) but still observed a significant reduction in *IGFBP-2* gene expression in LNCaP cells treated with metformin in both glucose conditions (Fig. 7A) indicating that, as in the DU145 cells, the effect of metformin on *IGFBP-2* expression was independent of AMPK. Successful inhibition of phosphorylation of AMPK with CC was confirmed by Western immunoblotting (Fig. 7A, insert). Furthermore, silencing both catalytic subunits AMPK α1 and α2 with siRNA did not abrogate metformin-induced inhibition of *IGFBP-2* gene expression (Fig. 7B). Metformin could still reduce *IGFBP-2* gene expression despite silencing *AMPK* suggesting an AMPK-independent mechanism of metformin action. Successful knockdown of AMPK was illustrated by Western immunoblot (Fig. 7B, insert). Consistent with this metformin could still reduce IGFBP-2 levels even in the presence of the AMPK inhibitor, Compound C (Supplementary Fig. 1A, see section on supplementary data given at the end of this article).

Having established that the impact of metformin on IGFBP-2 occurred independently of AMPK, we then examined whether the survival effect of high glucose was still present in LNCaP cells treated with Docetaxel, despite having silenced AMPK (Fig. 7C). With LNCaP, hyperglycaemia conferred resistance to Docetaxel in the presence of AMPK (12.4% difference between normal and high glucose level media) or with AMPK silenced (difference reduced to 6.4%, which was still significant).

In contrast, silencing IGFBP-2 negated high glucose-mediated survival of LNCaP cells treated with Docetaxel or metformin alone. In high glucose conditions, when *IGFBP-2* expression was enhanced neither Docetaxel nor metformin could induce cell death, but with *IGFBP-2* expression silenced, both could induce cell death, but there was no additive effect with the combination of metformin and Docetaxel (Fig. 7D).

Furthermore, the presence of the AMPK inhibitor, Compound C did not impact on the survival effect of high glucose in LNCaP cells treated with Docetaxel or metformin suggesting that AMPK is not a predominant pathway of metformin action (Supplementary Fig. 1B). Western blot shows that despite the small effect of CC in increasing the abundance of IGFBP-2, the opposite effect of metformin on IGFBP-2 protein was more robust (Supplementary Fig. 1A).

## Discussion

During recent years, there has been an increasing amount of evidence suggesting that diabetes is associated with increased incidence and/or progression of cancer and correlates with poor prognosis and survival for patients suffering from pancreatic (Everhart & Wright 1995, Huxley *et al*. 2005), colon (Larsson *et al*. 2005), kidney (Larsson & Wolk 2011) or breast cancer (Larsson *et al*. 2007). Meta-analysis of population-based studies, however, implied an opposite finding with prostate cancer, with diabetes acting as a protective factor against the risk of developing PCa (Kasper & Giovannucci 2006). A positive association between testosterone and prostate cancer was previously observed (Gann *et al*. 1996). The fact that diabetes and insulin-resistance decreases the levels of testosterone may relate this a negative association of PCa and T2D (Meyer *et al*. 2010). Diabetes is often accompanied with co-morbid conditions like obesity. Observational studies confirmed that obesity contributes to an increased aggressiveness of prostate cancer (Rodriguez *et al*. 2007, Su *et al*. 2011, Capitanio *et al*. 2012).

These data clearly suggest a negative impact of impaired metabolic status on prostate cancer progression. Our previous study evaluated the effects of elevated glucose levels on the ability of prostate cancer cell lines to respond to chemotherapy. Hyperglycaemia inhibited the efficacy of Docetaxel in inducing apoptosis of DU145 and LNCaP but not PC3 cell lines (Biernacka *et al*. 2013).

A common drug used to improve insulin resistance-associated glycaemic disturbances is metformin. This study investigated the effect of metformin on chemotherapy-induced cell death of prostate cancer cell lines and suggests a novel underlying mechanism. With DU145 and LNCaP PCa cells, we observed increased cell death in response to metformin alone in euglycaemic conditions, and the effect was reduced in hyperglycaemic conditions. In contrast, metformin has less of an effect on PC3 or VCaP cells, and hyperglycaemia was also without effect on the response of these cells. These data are consistent with other *in vitro* studies where metformin dose dependently (1 mM and 5 mM) decreased the ability LNCaP and DU145 cells to form colonies in soft agar (Ben Sahra *et al*. 2008). The same study indicated that LNCaP xenografts in diabetic mice treated with metformin had significantly reduced tumour growth (Ben Sahra *et al*. 2008). Colquhoun and coworkers used PC3 cells and with similar doses of metformin and found that metformin did not inhibit colony survival: these results are in keeping with our data showing that PC3 cells are much less sensitive to metformin treatment under euglycaemic and hyperglycaemic conditions (Colquhoun *et al*. 2012). Metformin absorption in human hepatocytes is facilitated by an organic cation transporter 1 (OCT1) (Shu *et al*. 2007). However, PC3 cells have abundant levels of OCT1 (Robles *et al*. 2002); therefore, this reduced sensitivity to metformin was clearly not limited by the uptake of metformin into PC3 cells. On examining the impact of metformin on the response of PCa cells to chemotherapy, we found that with DU145 cells, the hyperglycaemia-induced reduction in response to Docetaxel or metformin alone were abolished in the presence of both drugs and a similar pattern was observed in LNCaP cells. Interestingly, the combined treatment also stimulated an increase in cell death in both 5 mM and 25 mM glucose conditions. This suggests a beneficial effect of metformin usage for patients with normal or disturbed metabolic conditions such as diabetes. Epidemiological data indicate that prostate cancer patients who also suffer from diabetes have better outcomes when their diabetes is being treated with metformin (Wright & Stanford 2009, Morden *et al*. 2011). This indicates that Docetaxel and metformin together could markedly benefit PCa patients with or without co-existing diabetes. Interestingly, a study of 1734 men after radical prostatectomy or radiotherapy for localised PCa showed that out of ‘ever’ metformin users (*n* = 366), 143 (21%) patients taking metformin before and after diagnosis had decreased risk of prostate cancer biochemical recurrence (HR 0.55, 95% CI 0.31–0.96). In addition, when examining prostate needle biopsies from a subgroup, the use of metformin was associated with higher cytoplasmic staining of IGF-1R in men with PCa (Winters *et al*. 2015). This suggested that use of metformin could modify the IGF signalling pathways and potentially influence the development of PCa.

There is an increasing interest in understanding how metformin exerts its antineoplastic activity. It was most commonly thought that metformin acted via activation of AMPK, which is a central energy-sensing system of the cell, and that it did this via a separate kinase, LKB1 (Zhuang & Miskimins 2008). Once activated, AMPK inactivates a number of anabolic enzymes (such as fatty acid synthase; FASN) involved in ATP-consuming events such as fatty acid and protein synthesis and activates ATP-generating pathways like fatty acid oxidation (Foretz *et al*. 1998, Algire *et al*. 2010). Activated AMPK can control cell growth and proliferation by inhibiting mammalian target of rapamycin (mTOR) activity (Wullschleger *et al*. 2006). This results in an inhibition of proteins synthesis and proliferation.

We investigated the mechanism underlying the ability of metformin to induce cell death of prostate cancer cells. When screening PCa cell lines for LKB1 protein abundance, we found that among DU145, PC3, LNCaP and VCaP cells, only DU145 cells were LKB1 negative. We then examined the impact of metformin on AMPK activation. As anticipated, with DU145 we did not observe an increased phosphorylation of AMPK, which is in keeping with an involvement of LKB1 in AMPK activation. However, with LNCaP cells, there was a metformin-induced AMPK activation at threonine 172, which is thought to be phosphorylated by LKB1. Our findings show that metformin could induce cell death in both DU145 and LNCaP cells, suggesting that metformin can act in a LKB1–AMPK-independent way to induce cell death. This is not in agreement with studies that imply that LKB1 is essential for metformin-induced cell growth inhibition. Cell growth was not inhibited in LKB1-negative HeLa cells or MDA-MB-231 breast cancer cells in contrast to the inhibition observed with MC7, MCF10a and PC3 prostate cancer cells (Zakikhani *et al*. 2006). Others, however, have reported that metformin was able to reduce tumour growth mediated by high-fat diet and high glucose regardless of LKB1 expression *in vivo* (Algire *et al*. 2011). In addition, specific silencing of the *AMPK* pathway using siRNA to both catalytic subunits of AMPKα did not prevent the antiproliferative effect of metformin on PCa cell lines as well as reduce tumour growth *in vivo* in mouse LNCaP xenografts (Ben Sahra *et al*. 2008). These findings are in keeping with our results and show that LKB1 and activation of AMPK are not necessary for metformin to exert its anti-tumourigenic activity.

Our previous paper showed that the survival effect of hyperglycaemia was mediated by increased IGFBP-2 that can act in both, an IGF-IR-dependent manner in PC3 cells and intrinsically (independent of IGF interaction) in DU145 and LNCaP cells (Uzoh *et al*. 2011). IGFBP-2 is an established mitogen and survival factor for PCa (Chatterjee *et al*. 2004, Uzoh *et al*. 2011). Based on these findings, we have investigated a role of IGFBP-2 in relation to metformin actions. Metformin induced a decrease in *IGFBP-2* mRNA levels in DU145 and LNCaP, but not in VCaP cells. This was observed in both 5 mM and 25 mM glucose concentrations. The metformin-induced decrease in *IGFBP-2* gene expression in DU145 and LNCaP cells was accompanied with a reduction of IGFBP-2 protein from cell lysates as well as IGFBP-2 secreted into the media. There was no effect of metformin on IGFBP-2 protein and mRNA expression in VCaP cells. The pro-apoptotic effect of metformin was diminished when the reduced expression of *IGFBP-2* was replaced by the addition of exogenous IGFBP-2 in DU145 cells. Bearing in mind that DU145 cells do not express the LKB1 protein and that AMPK was not activated with metformin, we suggest that the LKB1-AMPK-independent action of metformin was, at least in part, via downregulation of the survival factor IGFBP-2. We published previously that high glucose induced the upregulation of IGFBP-2 that was associated with an increase in the levels of acetylated histones H4 and H3 associated with the *IGFBP-2* gene in DU145 and LNCaP cells (Biernacka *et al*. 2013). Our study indicates that metformin downregulates IGFBP-2 to negate the chemoresistance induced by high glucose: it will be interesting to determine if epigenetic modification of IGFBP-2 is a mechanism by which metformin is able to regulate IGFBP-2 levels.

In addition to the ability of metformin to phosphorylate AMPK in LKB1-positive LNCaP cells, we have also observed that metformin was also able to reduce gene and protein expression of IGFBP-2. Investigation at the mRNA level of *IGFBP-2* showed that metformin was still able to downregulate *IGFBP-2* gene after silencing *AMPK* using siRNA or inhibiting AMPK with Compound C. In addition, silencing *AMPK* did not alter the protective effect of high glucose in reducing Docetaxel-mediated cell death, suggesting that inhibition of AMPK did not influence the ability of IGFBP-2 to act as a survival factor in LNCaP cells. Furthermore, after knockdown of *IGFBP-2*, exposure to 25 mM glucose was no longer protective against Docetaxel or metformin treatment in relation to apoptosis suggesting that these protective effects were mediated by IGFBP-2. In addition to that, metformin was also able to exert its action in high and low glucose media even in the presence of Compound C again indicating this effect was not dependent on the activation of AMPK.

These findings suggest that metformin could induce cell death independently of the presence of LKB1 and without the involvement of AMPK but facilitated by the reduction of IGFBP-2 (a survival factor for PCa cells). This suggests that a novel potential mechanism of the anti-cancer effects of metformin may be by the regulation of IGFBP-2.

As members of the kinesin family have previously been shown to play a role in mediating taxane resistance in prostate cancer (Tan *et al*. 2012), we investigated if the kinesins most implicated in prostate cancer (Eg5 and KIF3a) were involved in our model of hyperglycaemia-induced chemoresistance. Our preliminary investigations did not provide any evidence that either Eg5 or KIF3a played a role; however, further work would be required to definitively eliminate the possible involvement of these and other members of this large family of kinesins.

Recently, there has been debate as to how data from *in vitro* studies of metformin translate into *in vivo* activity in animal models and clinical trials, as the majority of work *in vitro* has been performed using concentrations varying from 1 to 100 mM, predominantly between 1 and 20 mM (Dowling *et al*. 2007, Ben Sahra *et al*. 2008, Zakikhani *et al*. 2008, Hwang & Jeong 2010, Colquhoun *et al*. 2012, Lau *et al*. 2014). Such concentrations exceed levels measured in the blood of diabetic patients (Frid *et al*. 2010), although many organs like liver, kidney or small intestine are exposed to much higher concentration of metformin compared with levels measured in blood (Owen *et al*. 2000). Importantly, plasma membrane monoamine transporter (PMAT) or equilibrative nucleoside transporter ENT-4 facilitates metformin absorption from the lumen (Zhou *et al*. 2007). Other studies show the importance in expression of the OCT family to build up intracellular levels of metformin (Wilcock & Bailey 1994, Shu *et al*. 2007), and blocking OCT-1, -2 and -3 inhibited OCT-mediated transport of metformin (Nies *et al*. 2011). There is little evidence for the actual physiological concentrations of metformin achieved in normal and prostate cancer tissues although clearly metformin could accumulate over time.

The effect of metformin alone on the induction of cell death was much greater in normal glucose conditions and diminished in hyperglycaemia, but co-treatment with metformin and Docetaxel resulted in synergistic or additive effects and negated the hyperglycaemia-mediated reduction in sensitivity to Docetaxel. Investigating potential mechanisms through which metformin can act, we found that the presence of LKB1 and activation of AMPK were not required. In cells where metformin acted and potentiated the response to Docetaxel, this was associated with a reduction in the expression of *IGFBP-2* and a loss of its prosurvival actions.

## Supplementary data

This is linked to the online version of the paper at http://dx.doi.org/10.1530/ERC-16-0095.

## Declaration of interest

The authors declare that there is no conflict of interest that could be perceived as prejudicing the impartiality of the research reported.

## Funding

The authors thank the European Foundation for the Study of Diabetes (EFSD) and Bristol Urological Institute (BUI) for supporting the work. In addition, J M P H is supported by Cancer Research UK (C18281/A19169) Programme Grant (the Integrative Cancer Epidemiology Programme) and by the National Institute for Health Research (NIHR) Bristol Nutritional Biomedical Research Unit based at University Hospitals Bristol NHS Foundation Trust and the University of Bristol.
